# Genetics and Traumatic Brain Injury: Findings from an Exome-Based Study of a 50-Patient Case Series

**DOI:** 10.3390/cimb46090616

**Published:** 2024-09-17

**Authors:** Alesya S. Gracheva, Darya A. Kashatnikova, Ivan V. Redkin, Vladislav E. Zakharchenko, Artem N. Kuzovlev, Lyubov E. Salnikova

**Affiliations:** 1 The Department of Population Genetics, Vavilov Institute of General Genetics, Russian Academy of Sciences, 119991 Moscow, Russia; palesa@yandex.ru; 2 The Laboratory of Clinical Pathophysiology of Critical Conditions, Federal Research and Clinical Center of Intensive Care Medicine and Rehabilitology, 107031 Moscow, Russia; artem_kuzovlev@mail.ru; 3 The Laboratory of Ecological Genetics, Vavilov Institute of General Genetics, Russian Academy of Sciences, 119991 Moscow, Russia; daria_sv11@mail.ru; 4 The Laboratory of Molecular Pathophysiology, Lopukhin Federal Research and Clinical Center of Physical-Chemical Medicine of Federal Medical Biological Agency, 119435 Moscow, Russia; 5 The Laboratory of Organoprotection in Critical Conditions, Federal Research and Clinical Center of Intensive Care Medicine and Rehabilitology, 107031 Moscow, Russia; iredkin@fnkcrr.ru; 6 The Department of Clinical Laboratory Diagnostics, Federal Research and Clinical Center of Intensive Care Medicine and Rehabilitology, 107031 Moscow, Russia; vzakharchenko@fnkcrr.ru; 7 The Laboratory of Molecular Immunology, National Research Center of Pediatric Hematology, Oncology and Immunology, 117997 Moscow, Russia

**Keywords:** moderate/severe traumatic brain injury (msTBI), exome sequencing, rare high-impact (HI) variants, intolerant genes, nervous system disease-related genes

## Abstract

Traumatic brain injury (TBI) is the leading cause of global mortality and morbidity. Because TBI is accident-related, the role of genetics in predisposing to TBI has been largely unexplored. However, the likelihood of injury may not be entirely random and may be associated with certain physical and mental characteristics. In this study, we analyzed the exomes of 50 patients undergoing rehabilitation after TBI. Patients were divided into three groups according to rehabilitation outcome: improvement, no change, and deterioration/death. We focused on rare, potentially functional missense and high-impact variants in genes intolerant to these variants. The concordant results from the three independent groups of patients allowed for the suggestion of the existence of a genetic predisposition to TBI, associated with rare functional variations in intolerant genes, with a prevalent dominant mode of inheritance and neurological manifestations in the genetic phenotypes according to the OMIM database. Forty-four of the 50 patients had one or more rare, potentially deleterious variants in one or more neurological genes. Comparison of these results with those of a 50-sampled matched non-TBI cohort revealed significant differences: P = 2.6 × 10^−3^, OR = 4.89 (1.77–13.47). There were no differences in the distribution of the genes of interest between the TBI patient groups. Our exploratory study provides new insights into the impact of genetics on TBI risk and is the first to address potential genetic susceptibility to TBI.

## 1. Introduction

Traumatic brain injury (TBI) is transient or persistent brain dysfunction caused by an external mechanical force to the head. TBI is the leading cause of death, health loss, and disability worldwide, with various estimates of the annual global incidence of TBI ranging from 27 million to 69 million cases [1]. Because of its contribution to global death and trauma-related disability, it is often referred to as a “silent epidemic” [2]. The likelihood and degree of recovery and level of residual disability after moderate to severe (ms) TBI have been reported to vary widely [3,4]. In the long term, msTBI is associated with an increased risk of poor health, cognitive decline, and neurodegenerative disease, and an increased need for lifelong personal care [5,6].

Over the past 25 years, genetic discoveries have allowed us to better understand the mechanisms underlying many rare and common diseases, including TBI [7]. The major efforts of TBI geneticists have focused on finding genetic factors associated with recovery from TBI, particularly the likelihood, rate, and extent of recovery; the development and severity of post-traumatic stress disorder; and neurodegenerative diseases [8,9,10]. Brain repair after TBI may be strongly influenced by deleterious variants in neurodevelopmental genes. It is hypothesized that patients with such mutations who do not exhibit a developmental phenotype may be impaired in adult recovery processes where the environment is less favorable for plasticity [11]. Genes implicated in neurological dysfunction could also potentially increase the risk of poor response not only to msTBI but also to milder head trauma [12]. Because trauma is the result of an accident, the role of genetics in the likelihood of trauma itself has been virtually unstudied. However, the likelihood of injury may not be entirely random. Avoidance of injury is related to intelligence [13], a trait that has been shown to have a genetic component [14]. Traits associated with an increased likelihood of TBI, such as risky behavior and mental illness [15], reaction time, perceptual quality, and motor coordination, also have a genetic background [16,17,18], so TBI can be considered a multifactorial condition that includes a genetic factor.

Given that certain nervous system-related traits may predispose to TBI and the existing gap in the problem of potential genetic predisposition to TBI, we performed whole-exome sequencing in a series of 50 post-msTBI patients to investigate genetic factors statistically associated with (1) TBI onset and (2) TBI recovery. In contrast to array-based GWAS, exome sequencing allows for the discovery of rare variants, and we focused on highly penetrant rare genetic variants, which are known to have large effect sizes and may be of great importance in the context of the problem discussed [9,19]. In addition, exome sequencing can detect de novo variation, which on average has been exposed to less selective pressure and may have more severe predicted functional consequences than inherited variation [20]. To reduce dimensionality, we further focused on the role of potentially pathogenic variants in genes that are intolerant to these variants. As the patients were undergoing rehabilitation, our second task was to try to assess the role of the variants and genes under study in groups of patients with different rehabilitation outcomes. In the final phase of the study, we performed a genetic analysis in 50 non-TBI patients to assess the biological relevance of the results obtained. Thus, in this exploratory study, we used a novel approach to test the hypothesis of the role of rare functional variants of intolerant genes in general and nervous system-related intolerant genes in particular in patients with msTBI in both predisposition to and recovery from TBI.

## 2. Materials and Methods

### 2.1. Participants

Participants were recruited from patients undergoing rehabilitation after TBI at the Federal Research and Clinical Center of Intensive Care Medicine and Rehabilitology (Moscow, Russia) between June 2023 and May 2024. The study included 50 unrelated patients with a history of msTBI, representing a wide range of patients who received initial treatment in different clinics and were hospitalized for rehabilitation at different times (from 9 to 526 days) after the accident. Patients were enrolled if they were older than 17 years and had confirmed TBI, regardless of the presence or absence of a background disorder due to brain damage and dysfunction. Exclusion criteria were as follows: patients with psychiatric disorders (e.g., schizophrenia or bipolar disorder), neurological disorders (e.g., stroke, tumor), developmental disorders, inherited neurological and central nervous system diseases, pregnancy, terminal chronic diseases, alcohol- or drug-related injuries, and war injuries. The prevalence of traumatic experiences in patients with mental illness [21,22] and developmental disabilities [23] is well documented. In addition, treatment outcomes may differ between individuals with and without psychiatric disorders [24]. To assess potential susceptibility to TBI per se, rather than psychiatric/neurological disorders, and to eliminate the influence of these primary conditions on recovery from TBI, our exclusion criteria, although reducing the potential applicability of the results, were designed to remove potential confounders in the study of genetic susceptibility to TBI and various TBI outcomes after rehabilitation. The study protocol (#2/23/4, 30 May 2023) was approved by the Institutional Review Board (IRB) of the Federal Research and Clinical Center of Intensive Care Medicine and Rehabilitology. Written informed consent was signed by all enrolled patients or their legal representatives.

Rehabilitation was performed according to approved protocols and included early rehabilitation (if necessary) and rehabilitation treatment. The main procedures of early rehabilitation are positioning treatment, passive and active gymnastics, massage, weaning from artificial ventilation (vening), spontaneous breathing training, coughing training, verticalization, swallowing diagnostics and training, speech therapy, phoniatrics, neuropsychology, pedagogical courses, functional electrical stimulation, use of simulators, and thoracic vibromassage. The main procedures of rehabilitation treatment (in addition to the early rehabilitation complex) are physiotherapy, hydrokinesiotherapy and kinesiotherapy, methods based on biofeedback, virtual and augmented virtual reality, neurocomputer interface, electronic assistive technologies and robotic methods, occupational therapy, and others.

Patients were divided into three groups according to outcome: improvement (*n* = 25), no change (*n* = 18), and deterioration/death (*n* = 7). Outcome measures included comparisons between the patient’s condition at the first and last assessments before discharge, transfer, or death based on commonly used brain injury assessment tools such as the Glasgow Coma Scale (GCS), Coma Recovery Scale-Revised (CRS-R), Disability Rating Scale (DRS), and Full Outline of UnResponsiveness (FOUR) scale [25,26,27,28] (Table S1). Validation and comparative analysis of the scales are available in many studies [29,30,31]. The Sepsis-related Organ Failure Assessment (SOFA) scale (Table S1), which quantifies the number and severity of failing organs [32], was used to assess the presence of critical illness. Neurological scales were not the only criterion for determining a favorable outcome. The assessment of rehabilitation outcomes was determined by consensus among the various specialists involved in rehabilitation. In general, a favorable outcome (group 1) was indicated by a moderate improvement on 3–4 scales, i.e., an improvement in the overall assessment results within the accepted gradation (Table S1), or a significant improvement approaching optimal values on one or two scales, with no deterioration on other scales. Patients in group 2 showed no improvement either in the neurological scale scores or in the expert opinion. In group 3, five patients died as a result of septic shock and two patients were transferred to other medical yards by the decision of the medical committee due to deterioration of physical condition followed by worsening of neurological scale scores.

To validate our findings in an independent non-TBI cohort with available medical history and WES data, we used our previously described cohort of COVID-19 patients [33]. From the sample of 86 patients, we selected individuals using the same criteria as for TBI patients, taking into account the need to match by age and sex. The sample of non-TBI patients included 32 males and 18 females with an age of 51.14 ± 16.28 years (mean ± SD). Thus, our non-TBI sample was methodologically convenient, providing a matched and balanced case–control study. With regard to the selection of controls to ensure the validity and power of clinical research, the control group should consist of individuals who are similar to the subject group in all aspects that affect the outcome except for the condition of interest [34]. In our study, the condition of interest was TBI with severe sequelae requiring specialized daily care. There were no such patients among our COVID-19 patients, according to medical histories, consistent with the fact that patients were admitted to standard infectious disease units of urban hospitals.

### 2.2. Exome Sequencing and Variant Calling

Blood was isolated using the QIAamp DNA Blood Mini Kit according to the manufacturer’s protocol. Samples were sequenced at LLC “Eugene” (Moscow, Russia). DNA was mechanically fragmented using an S220 Focused-ultrasonic disintegrator (Covaris) to an average fragment length of 250 bp. Enrichment was performed by hybridization technology using the Agilent SureSelect Human All Exon v8 probe set (target size—at least 35 million bp) according to the RSMU_exome protocol [35]. Pools of enriched DNA libraries were circularized and sequenced in paired-end mode on the MGISEQ-2000 platform using the DNBSEQG400RS high-throughput sequencing kit PE100 according to the manufacturer’s protocol (MGI Tech). FastQ files were generated using the manufacturer’s zebracall-V2 software (MGI Tech). Bioinformatic processing of the sequencing data for each sample included an alignment procedure for the GRCh37 reference genome using bwa mem2 v2.2.1 and SAMtools v1.9, obtaining quality metrics for exome enrichment using Picard v2.22.4 software, variant calling using bcftools mpileup v1.9 and Strelka2 v2.9.2 software, variant annotation using the web version of ANNOVAR software (https://annovar.openbioinformatics.org/en/latest/, accessed on 17 June 2024) [36], and a set of proprietary scripts to optimize and improve the quality of the content of the final variant annotation files [35]. Upon completion of the bioinformatics pipeline, the MultiQC v1.14 software was run as a final quality control step. Variant calls were required to have at least 10× coverage. The average sequencing depth was 116.96 ± 55.05 (mean ± SD).

### 2.3. Variant Annotation

Variant annotation was performed using the AnnoVar and Ensembl Variant Effect Predictor (VEP) (release 112) tools, including in particular the conversion of the hg19 to hg38 human genome versions, obtaining allele frequency (AF) data from the Genome Aggregation Database (GnomAD) and clinical signature data from ClinVar. Variants with a putative disruptive effect on the protein were classified as high impact (HI). These included splice acceptor, splice donor, stop gained, frameshift, stop lost, and start lost variants. Missense variants were defined as potentially pathogenic using the Rare Exome Variant Ensemble Learner (REVEL) tool with a recommended threshold of >0.5 to classify the variant as “deleterious” [37]. Our analysis focused on rare variants, which were filtered by alternative AF in GnomAD v2.1.1. Variants with AF < 0.001 and no AF data in GnomAD and with no more than 3 alleles in our sample were considered rare. We generated sets of genes intolerant to missense variants and loss of function, i.e., HI variants, using the recommended thresholds of pLI > 0.9 and missense Z-score > 3.09 based on the constraint metrics from gnomAD v2.1.1 (https://gnomad.broadinstitute.org/downloads, accessed on 27 June 2024). Rare HI and missense variants (REVEL > 0.5) in intolerant genes, called qualifying variants (QVs), comprised the main set of variants considered, which were additionally checked for VarSome [38] annotations.

### 2.4. Data Analysis

Gene function enrichment analyses of gene lists with rare functional variants were performed with Enrichr (https://maayanlab.cloud/Enrichr/, accessed on 15 June 2024) using the Reactome 2022 and GO Biological Process 2023 modules. FDR-adjusted *p*-values < 0.05 were considered statistically significant. Additional analyses included the categorization of Reactome pathways into Reactome top-level pathways (https://reactome.org/download-data, accessed on 15 June 2024) and GO list reduction using REVIGO (http://revigo.irb.hr/, accessed on 16 June 2024), which simplifies lists by grouping terms with similar functions based on semantic similarities. For the analysis of intolerant genes with QVs, we used (i) DOMINO to infer the mode of inheritance of candidate genes (https://wwwfbm.unil.ch/domino/, accessed on 16 June 2024); (ii) OMIM Gene Map to retrieve all genes with phenotype-causing mutation (https://www.omim.org/search/advanced/geneMap, accessed on 18 June 2024) and Clinical Synopsis, with the entry “neurologic” (https://www.omim.org/search/advanced/clinicalSynopsis, accessed on 18 June 2024) to further select genes associated with neurological manifestations; (iii) the Reactome Pathways Gene Set module (https://reactome.org/download-data, accessed on 15 June 2024) to retrieve all pathways for the genes of interest; (iv) the AmiGO 2 tool (https://amigo.geneontology.org/amigo/landing, accessed on 18 June 2024) to retrieve genes associated with “nervous system process” (GO:0050877), the top nervous system-related term within the “Biological Process” domain in taxon 9606 Homo sapiens; and (v) GnomAD v.2.1.1 9 (non-neuro), which contains 114,704 samples from individuals who have not been found to have a neurological condition in a neurological case–control study to control for the presence of rare HI variants in OMIM genes associated with neurological manifestations. Only loss of function, i.e., HI variants with AF < 0.001 that passed the exome filters and were not flagged, were included.

Statistical analysis was performed with R software (version 3.4.1). Because the groups compared were small, for categorical variables we used Fisher’s exact chi-squared test with Yates’ continuity correction to test whether the proportion of the variable in question was the same in the samples compared. Groups were compared in pairs, taking into account the likelihood of making pairwise comparisons of genetic study results. Yates’ correction was used to avoid overestimating statistical significance for small data (at least one cell of the table has an expected count of less than 5). For continuous variables, we first tested the normality of the distribution using the Shapiro–Wilk test. The normality test revealed violations of normality for a portion of the data samples, so to test whether the compared samples were pooled from the same sample, we next used the nonparametric Kruskal–Wallis test (for a three-group comparison) with the Nemenyi post hoc test to identify specific group differences and the Mann–Whitney U (MWU) test (for a two-group comparison). Pearson’s correlation coefficients (assumed when data follow a normal distribution) and Spearman’s correlation coefficients (assumed when data do not follow a normal distribution) were used to describe the strength and direction of the relationship between compared variables. For multiple comparisons (neurological scale data for pre- and post-rehabilitation scores, the results of enrichment analyses, and the results of gene-based comparison of TBI and non-TBI patients), FDR-adjusted *p*-values < 0.05 were considered statistically significant.

Given the study design, sample power was not estimated. The vast majority of variants studied were singletons with unknown allele frequencies and likely varying effect sizes. This study design involves many unknown parameters that must be modeled to evaluate sample power, making power analysis complicated and impractical [39]. In addition to statistical significance in the direct comparison of patients with and without TBI under a balanced case–control scenario [40], the reliability of the study results was improved by enrichment analysis in the whole group and patient subgroups [41], and dimensionality reduction was achieved by estimating the cumulative frequency of rare functional variants in intolerant genes. However, due to the small sample size, all results are considered preliminary.

Graphs were generated using https://www.bioinformatics.com.cn/srplot (accessed on 20 June 2024), a free online platform for data analysis and visualization [42], and PhenoGram software (https://ritchielab.org/software/phenogram-downloads, accessed on 20 June 2024) [43].

## 3. Results

### 3.1. Patients

Our case series included 50 unrelated patients with sequelae msTBI who were admitted to rehabilitation at various times after the accident and initial treatment (Table 1). On admission, nine patients had Glasgow Coma Scale (GCS) scores consistent with severe TBI (scores of 3–8), 22 patients had moderate TBI (scores of 9–12), and the remainder had partial recovery and mild symptoms (scores of 13–15) (Table S1). The pattern of causes of TBI was generally consistent with global statistics, where falls account for nearly half of TBI-related hospital admissions (46% in our series), and motor vehicle accidents and assaults (28% and 6% in our series, respectively) are other common ways of sustaining TBI (https://www.cdc.gov/traumatic-brain-injury/data-research/facts-stats/; accessed on 9 July 2024). Most patients had closed TBI with various combinations of contusions, skull fractures, hemorrhages, and hematomas. Prior to admission to rehabilitation, 70% of patients had undergone surgery for acute post-TBI symptoms. Patients were divided into three groups according to outcome, i.e., improvement (group 1, *n* = 25), no change (group 2, *n* = 18), and deterioration/death (group 3, *n* = 7) (Table 1). The groups of patients did not differ with respect to sex, age, causes and types of TBI, surgical intervention, comorbidity, and clinical data.

**Figure 1:**
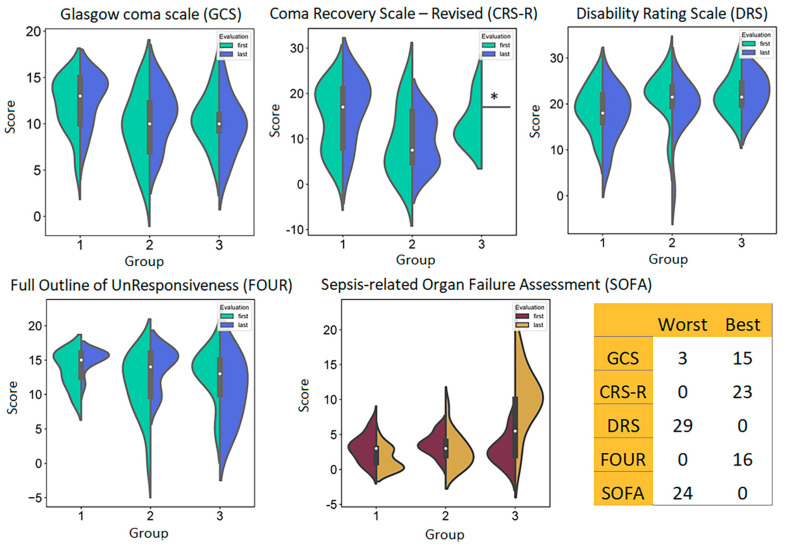
Violin plots for neurological scales and SOFA scale scores in three groups of patients at baseline and final measurements. The plots combine box plots common to both measurements (white dot is median) and kernel density plots showing the variation of the data across the distribution. Wider areas of the violin plot correspond to a higher probability that members of the sample will take the given value; the thinner areas correspond to a lower probability. The Kernel Density Estimation (KDE) plot smooths the distribution based on the trajectory of the data toward the convergence point. This explains values beyond the original min–max data. * In group 3, only one patient had a CRS-R score after treatment.

The within-group results of the pre- and post-rehabilitation scores using the brain injury assessment tools and the SOFA scale tool are summarized in Tables S1 and S2 and in Figure 1. In the total sample, significant correlations were found between the first and last measurement for the GCS and FOUR scores, although the correlation coefficient was less than 0.5 (Table S2). Some marginally significant differences between the first and last scores in group 2 versus group 1 became nonsignificant after FDR correction. For the SOFA scores, the differences between group 1 and group 3 were significant at the last measurement (*p* = 0.0004) (Table S2). Violin plots (Figure 1) representing the distribution of scores on the same panel for the first and last assessments show that in group 1, the distribution area corresponding to worse neurological assessments decreased after treatment compared to before treatment. For example, for the GCS, CRS-R, and especially the FOUR scales, where higher scores are better, the post-treatment scores tended to form density peaks (more patients with higher scores) at higher levels and to decrease in density at the lower extremes of the distributions. For the DRS scale, there was a small, smooth shift toward lower (better) final scores, indicating that some patients progressed in rehabilitation on this scale as well. In group 2, this effect was weaker or absent. It should be noted that patients in group 2 were more likely to have worse scores at both baseline and follow-up than those in group 1. Group 3 showed a trend in the distribution toward worse scores at follow-up on the DRS and FOUR scales. SOFA scores were similar in the three groups at baseline and remained similar at follow-up in groups 1 and 2, with the post-treatment bumps (higher densities) shifting toward better (lower) SOFA scores, i.e., lower likelihood of developing organ dysfunction. In group 3, where five of seven patients developed septic shock with organ failure, the final scores formed a bump at the worse (higher) levels of the scale, indicating a large bias toward an adverse outcome (Figure 1, Table S2).

### 3.2. Genetic Landscape Overview

**Figure 2:**
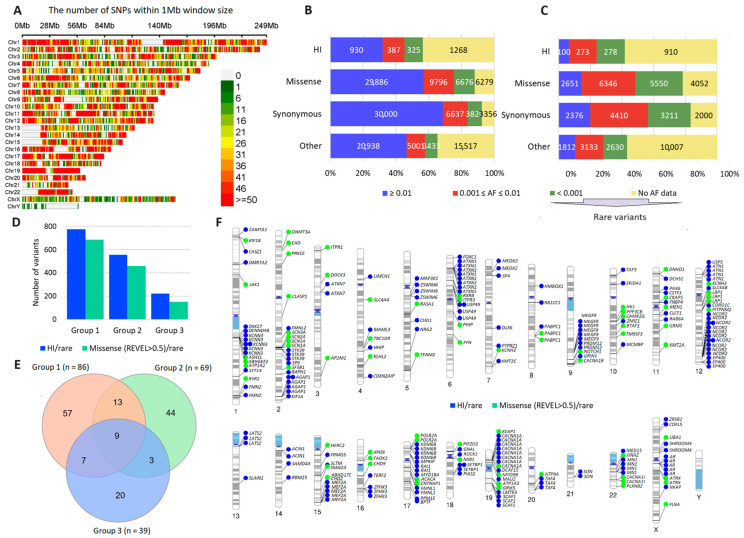
Exome data. (**A**) SNP density per chromosome. (**B**) A 100% stacked bar chart for the distribution of variants by allele frequency and functional consequences. (**C**) A 100% stacked bar chart for the distribution of variants by number of singletons. (**D**) Distribution of the number of HI/rare and potentially harmful rare missense variants (REVEL > 0.5) in the patient groups. (**E**) Venn diagram for the lists of intolerant genes with QVs (intolerant genes with HI/rare and missense (REVEL > 0.5)/rare variants in the patient groups. (**F**) Chromosome ideogram showing the cytogenetic band location of intolerant genes with QVs and the frequency of these genes in 50 patients.

A total of 1,740,233 genetic variants were identified in our sample; the number of variants per person was 34,804.66 ± 1119.61. The number of unique variants in the sample, i.e., variants with unique identifiers (position and alleles), was 144,249. These variants were located in 19,409 different genes. The SNP density distribution by chromosome is shown in Figure 2A. Gaps (light gray pieces) are fitted to gaps and issues of the recently published complete sequence of a human genome, T2T-CHM13 [44]. The distribution of variants by AF and different functional consequences is shown in Figure 2B. In general, common variants (AF > 0.01) were most frequent, with missense variants dominating among variants with different functional consequences. Among high-impact (HI) variants, variants without AF were the most common. The number of singletons (Figure 2C) was relatively low for variants with AF > 0.01 and was highest for rare (AF < 0.001/without AF) HI variants and other variants. The distribution of the number of HI/rare and potentially harmful rare missense variants (REVEL > 0.5) did not differ between the patient groups (Figure 2D).

Next, we performed the analysis of QVs, i.e., rare HI and rare potentially deleterious missense variants (REVEL > 0.5) in intolerant genes. Only one patient (group 1) had no QVs. Out of a total of 284 QVs, 282 were in a heterozygous state; 38 were reported in ClinVar, of which only one QV was reported to be likely pathogenic; and out of 84 QVs with VarSome verdict, 50 were reported to be pathogenic (strong, moderate, or supporting) (Table S3). Gene-based analysis of a total of 153 intolerant genes with QVs revealed a rather large overlap in the patient groups (Figure 2E). The chromosome ideogram showing the cytogenetic band location of intolerant genes with QVs and the frequency of unique QVs in these genes in 50 patients is presented in Figure 2F. Among the 153 genes with QVs, 34 genes with different QVs occurred more than once, the most frequent being *NCOR2*, chromosome 12q24.31 (*n* = 12); *CACNA1A*, chromosome 19p13.13 (*n* = 8); *ATXN1*, chromosome 6p22.3 (*n* = 8); and *KCNN3*, chromosome 1q21.3 (*n* = 6). The large gene overlap between patients seemed unexpected, since the number of HI variant-intolerant genes in GnomAD v.2.1.1 is 3110 and the number of missense variant intolerant genes is 969 (https://gnomad.broadinstitute.org/downloads, accessed on 27 June 2024).

### 3.3. Enrichment Analysis of Gene Function in the Whole Series of 50 TBI Patients

**Figure 3:**
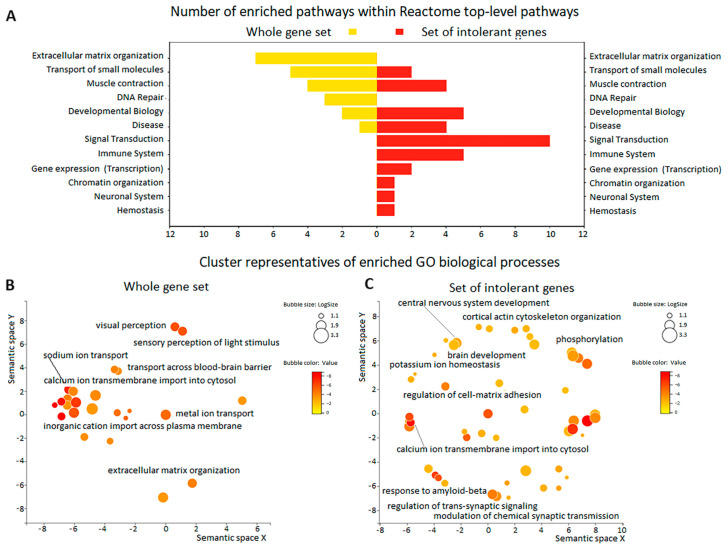
Enrichment analysis of genes of interest in the series of 50 TBI patients. (**A**) Dual Y-axis bar plot for the number of enriched pathways within Reactome top-level pathways for all genes with rare HI and missense variants (REVEL > 0.5) (left panel) and intolerant genes with these variants (right panel). (**B**,**C**) REVIGO scatterplots for the cluster representatives of enriched GO terms for genes with rare HI and missense variants (REVEL > 0.5) (**B**) and intolerant genes with these variants (**C**). Bubble color indicates *p*-value (legend top right); size indicates frequency of GO term in underlying GOA database (bubbles of more general terms are larger). Functionally similar GO terms are close together.

Comparison of the number of enriched pathways within the Reactome top-level pathways for genes with rare HI and missense variants (REVEL > 0.5) and for intolerant genes with these variants showed an increase in the number of pathways associated with Developmental Biology and Disease, and the appearance of new pathways, especially signal transduction, in the latter compared to the former (Figure 3A, Table S3). Interestingly, refinement of the developmental biology pathway classification revealed that it included nervous system development and axon guidance. The results of the GO BP enrichment analysis are consistent with and complementary to the results of the pathway enrichment analysis. GO terms for genes with rare HI and missense variants (REVEL > 0.5) were represented by terms related to transport and nervous system processes (sensory and visual perception) (Figure 3B, Table S4). Intolerant genes with these variants were enriched for GO terms related to nervous system development, transport, and synaptic signaling (Figure 3C, Table S4).

### 3.4. Enrichment Analysis of Intolerant Gene Function in the Three Groups of Patients with TBI

**Figure 4:**
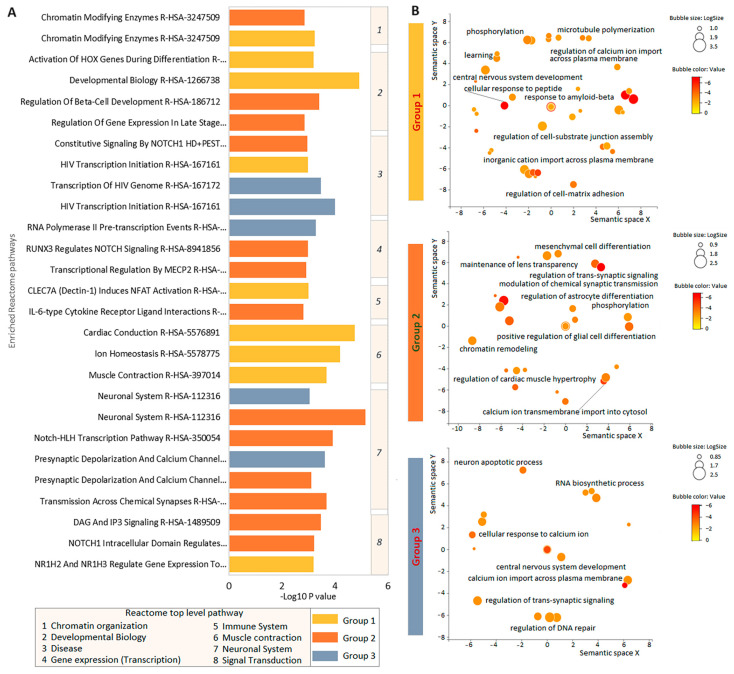
Enrichment analysis of intolerant genes in three groups of TBI patients. (**A**) Enriched Reactome pathways. The pathways are grouped into eight top-level Reactome pathways, which are indicated by numbers in the vertical beige column to the right of the bar graph. The correspondence of the number to the top-level Reactome pathways is shown in the legend at the bottom left. (**B**) REVIGO scatterplots for cluster representatives of enriched GO terms for gene sets in three patient groups. Bubble color indicates *p*-value (legend top right); size indicates frequency of GO term in underlying GOA database (bubbles of more general terms are larger). Functionally similar GO terms are close together.

Next, we performed Reactome and GO BP enrichment analysis for intolerant genes with QVs for three groups of patients. The largest number of enriched pathways within the top-level Reactome pathway was related to the neuronal system (*n* = 4 in group 2 and *n* = 2 in group 3) (Figure 4A, Table S5). Gene sets for all three patient groups were enriched for GO terms related to calcium ion transport as well as for some terms related to nervous system process, but in group 1 the corresponding term was more general, whereas in groups 2 and 3 there were more specific terms, such as terms related to synaptic signaling (group 2 and group 3), glial cell differentiation (group 2), and neuronal apoptotic process (group 3) (Figure 4B, Table S5).

### 3.5. Gene-Based Synopsis

**Figure 5:**
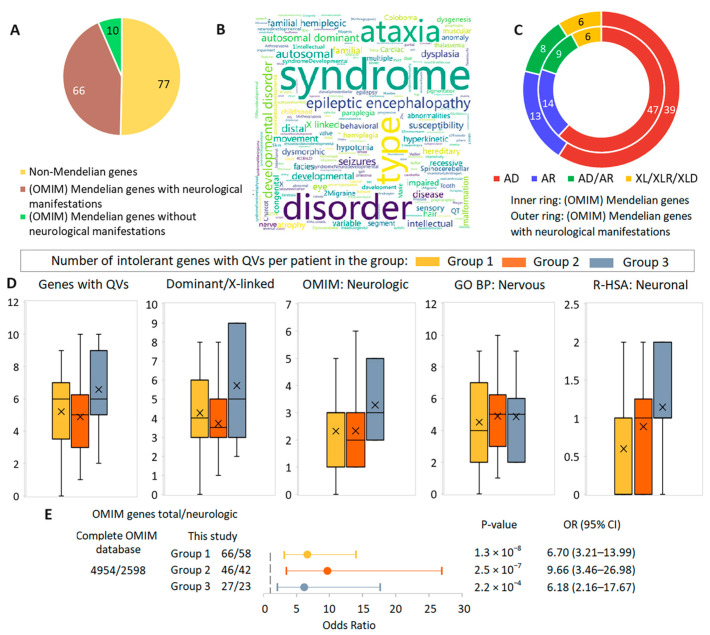
Overview of intolerant genes with QVs. (**A**) Pie chart for gene distribution according to OMIM phenotypes. (**B**) Word cloud for OMIM phenotypes associated with 76 genes. (**C**) Doughnut chart for OMIM gene distribution by mode of inheritance. (**D**) Box plots for the number of gene features considered per patient in the patient groups. (**E**) Comparison of the proportions of genes with neurological manifestations among all phenotype-associated genes in the OMIM database and in the patient groups. Odds ratios and horizontal bars indicating 95% confidence intervals are shown. (**D**,**E**) Complete gene sets were considered for each patient group (i.e., if a gene occurred in two patients, it was counted twice). R-HSA is a Reactome pathway.

Some characteristics of intolerant genes with QVs are given in Figure 5 and Table S6. Out of a total of 153 unique genes, 76 genes were associated with OMIM phenotypes (49.7%), among which those with neurological manifestations (*n* = 66, 86.8%) strongly predominated (Figure 5A). Forty-four out of 50 patients (88.0%) had one or more QVs in one or more nervous system disease-related genes. A word cloud of OMIM phenotypes for these genes demonstrated that the majority of phenotypes are severe inherited neurological syndromes (Figure 5B). Among the OMIM genes, those with AD-type inheritance accounted for 59.1%, and among the nervous system disease-related genes, 61.8%. To consider non-OMIM genes we used DOMINO-predicted metrics, and a very likely dominant type of inheritance was seen for 96 out of 153 genes (62.7%) (Table S6). The number of gene-based characteristics considered per patient in the patient groups showed no significant differences between them. In group 3, a trend towards higher values was observed for most of the characteristics compared to groups 1 and 2, but in group 3, five out of seven patients had septic shock, which adds another level of complexity to the interpretation of the results (Figure 5D). We also compared the distribution of specific gene sets with QVs per patient between male and female groups, but no differences were found (Table S6, Figure S1).

Next, we compared the number of genes associated with neurological manifestations among all phenotype-associated genes in OMIM and in the gene sets for each patient group. A significant overrepresentation of nervous system disease-related genes was observed in all three groups. The largest effect size was found in group 2 (Figure 5E). However, as the frequency of Mendelian disorders worldwide is variable, often unknown, or wide ranging, we view these results with some concerns regarding statistical estimates and suggest that they primarily reflect a similar distribution of Mendelian genes associated with nervous system disorders in the subgroups studied.

We continued our analysis assuming that the fact that almost half of the intolerant genes in our series are associated with OMIM phenotypes is a consequence of the applied study algorithm aimed at selecting specifically relevant genes, but the large percentage of genes with a dominant mode of inheritance seemed unexpected. We tested whether these results were consistent with the distribution of inheritance patterns in the full set of OMIM genes associated with phenotypes. Dominant inheritance was reported for 1516 out of 4954 metrics (30.6%); for the subset of genes with neurological manifestations, it was reported for 777 out of 2598 (29.9%). Thus, in our gene set, dominant inheritance was observed in a two-fold higher number of genes compared to the total sample of OMIM phenotype-associated genes.

Finally, for genes with QVs in >5 patients, we present summary information from OMIM, Reactome, and GO (Tables S4–S6), where available, that may provide a mechanistic link between these genes and TBI predisposition or outcome. QVs in *NCOR2* were found in 19 of 50 patients. *NCOR2* has no associations in the OMIM database. It encodes a protein involved in signal transduction (Reactome) and cerebellum development (GO). QVs in *CACNA1A* were met in 10 patients. *CACNA1A* mutations are associated with five nervous system-related OMIM phenotypes (Table S6). *CACNA1A* encodes a protein related to the neuronal system (Reactome), specifically transmission across chemical synapses, and to calcium ion transport and chemical synaptic transmission (GO). QVs in *ATXN1* were present in nine patients. Mutations in *ATXN1* are associated with the OMIM phenotype spinocerebellar ataxia 1. *ATXN1* is involved in nervous system development and social behavior according to GO, but has no data in Reactome. QVs in *KCNN3* were present in six patients. Mutations in *KCNN3* are associated with Zimmermann–Laband syndrome 3 (OMIM); the neuronal system, specifically potassium channels (Reactome); and potassium ion transport and neuron projection (GO). Two other genes, *AGAP1* and *MEGF9*, with QVs in six patients each, currently lack nervous system-related information in OMIM, Reactome, and GO, but recent articles have provided additional insight into the role of *AGAP1* and *MEGF9* in nervous system disorders [45,46].

### 3.6. Comparative Analysis of the Role of Rare Potentially Pathogenic Variants in Intolerant Genes in Other Cohorts in the Context of TBI Case Series Findings

According to the exclusion criteria, patients in our study did not have neurological and neurodevelopmental diseases. To confirm that rare HI variants in intolerant genes with neurological manifestations according to OMIM might not be associated with the neurological phenotype, we checked for the presence of variants of interest in the genes of interest (*n* = 66, see Figure 5A) in the GnomAD v.2.1.1 non-neuro set. We found that 64 of these 66 genes had such variants in the population dataset. The mean number of rare HI variants per gene was 18.69 ± 22.50, the mean allele count was 37.78 ± 53.62, and the mean AF was 1.06 × 10^−5^ ± 4.07 × 10^−5^ (Table S6).

**Figure 6:**
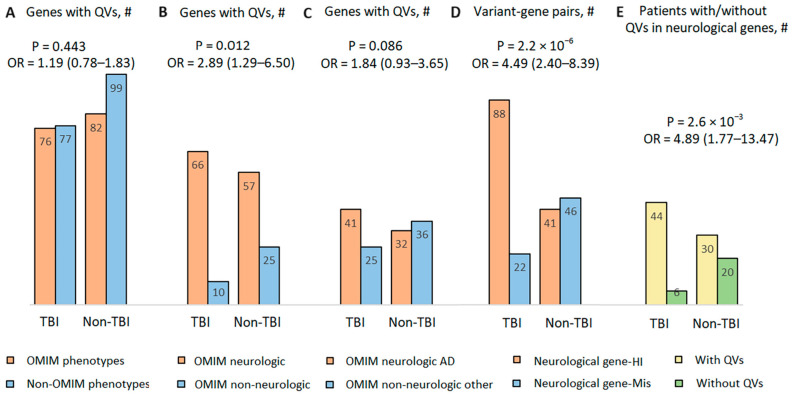
Comparison of the distribution of QVs in patients with and without TBI. (**A**) Number of genes with QVs that have an OMIM phenotype. (**B**) Number of OMIM neurological genes with QVs among all OMIM genes. (**C**) Number of OMIM neurological genes with AD mode of inheritance. (**D**) Number of neurological genes with damaging missense variants and with HI variants. (**E**) Number of patients with and without QVs in neurological genes. *p*-values in panels (**B**,**D**,**E**) are significant after FDR correction. The 95% confidence intervals for the odds ratio are given in parentheses.

Another cohort consisted of 50 age- and sex-matched non-TBI patients with whole-exome sequencing (WES) data from our previously described study of COVID-19 patients [33] (Table S7). Patients were selected using the same criteria as for TBI patients, and WES results were processed in the same manner as in the TBI study (Figure 6, Table S7). Of the total 181 unique genes, 82 genes were associated with OMIM phenotypes (45.3%) (Figure 6A), and among genes with OMIM phenotypes, those with neurological manifestations (*n* = 57, 69.5%) predominated (Figure 6B), although not as strongly as in TBI patients. There were no differences in the distribution of neurological genes with QVs in terms of mode of inheritance between TBI and non-TBI patients (Figure 6C). The largest differences were found when comparing gene–variant pairs, with more rare HI variants in neurological genes in TBI patients and more rare damaging missense variants in neurological genes in non-TBI patients (Figure 6D); odds ratio estimates OR = 4.49 (95% CI 2.40–8.39). The number of patients carrying one or more QVs in one or more neurological genes was higher in TBI patients compared to non-TBI patients (Figure 6E); odds ratio estimates OR = 4.89 (95% CI 1.77–13.47). Given the small sample size, we cautiously note that the relatively high odds for these two comparisons can be regarded as promising, as an odds ratio greater than 4 is considered relatively strong and has little chance of being explained by other unmeasured factors [47].

## 4. Discussion

In this study, we analyzed the exomes of 50 patients undergoing rehabilitation after msTBI. Patients were divided into three groups according to rehabilitation outcome: improvement, no change, and deterioration/death. We focused on rare HI and potentially deleterious missense variants (REVEL > 0.5) in genes that are intolerant to these variants. Enrichment analysis revealed that genes associated with calcium transport/homeostasis and nervous system development/function, including synaptic signaling and axon guidance, were overrepresented in the total set of intolerant genes and/or in the gene sets of the patient groups. The gene sets considered were also characterized by a high prevalence of nervous system disease-related genes and a dominant mode of inheritance according to the OMIM database. There were no significant differences in the distribution of intolerant genes between the patient groups. Analysis of QVs in neurological genes in two independent non-neuro cohorts (non-neuro GnomAD and non-TBI COVID-19 patients) provided two important findings: (1) supported the view that QVs may not be associated with the clinical neurological phenotype, and (2) supported the hypothesis of a potential genetic predisposition to TBI, as the TBI sample included more patients with HI variants in neurological genes compared to the non-TBI sample.

Biological processes and pathways affected by genetic variation in our study are associated with cognitive processes and neurodevelopmental or neurodegenerative diseases. Calcium (Ca^2+^) plays a fundamental role in neuronal plasticity. As a mediator in many signaling pathways, calcium has been shown to regulate neuronal gene expression, energy production, membrane excitability, synaptogenesis, and synaptic transmission, as well as neuroinflammation, neuronal injury, neurogenesis, neurotoxicity, neuroprotection, and autophagy [48,49]. Calcium dysregulation directly affects synapses (transmitter release, synaptic plasticity and integrity) and influences circuit outputs (sensory plasticity and network synchronization) [50]. Synaptic signaling is involved in communication between neurons, and genetic variations that disrupt synaptic proteins are associated with more than 130 brain disorders [51]. Neuronal guidance genes (encoding cues, receptors, or downstream transmitters) are essential not only for nervous system development but also for synaptic preservation, remodeling, and function in the adult brain [52]. Developmental and degenerative diseases of the nervous system are characterized by dysregulated calcium homeostasis and signaling, synapse loss, and other functional impairments [49,51]. In generally healthy individuals, calcium transport/homeostasis, synaptic signaling, and axon guidance are important for cognitive and motor functions such as learning, memory, attention, motor and sensory processing, and motor coordination [18,50,53,54]. Given the role of these processes and pathways in nervous system function, it is not surprising that perturbations resulting from genetic variation in the responsible genes lead to functional changes that affect individual mental, behavioral, and motor activity patterns in apparently normal individuals [11].

In light of the study results and the literature data, we propose the following hypothesis to explain the study observations. It is known that in the general population each individual carries a certain number of potentially deleterious variants (from 100 to 800) [55,56,57,58]. Some of these are rare and involve intolerant genes. The average genome contains 7.6 rare functional variants in monogenic disease genes [59], but only a minority of deleterious variants can actually cause a penetrant Mendelian disease [60]. The pathogenicity of variants depends on the specific genetic context [61], and variants vary in penetrance and expressivity. Individuals with the same genotype can have very different clinical phenotypes [62], including those who are clinically asymptomatic. Manifestations ranging from severe to clinically asymptomatic can be observed even in members of the same family who are carriers of the same variant [63,64]. In order to manifest at the phenotypic level, the functional consequences of a variant must not be compensated for along the lengthy pathway of realization of genetic information from the genomic level through the transcriptomic, proteomic and metabolomic, cellular, tissue, and physiological levels [65]. In some individuals of the general population, compensatory effects may be successful and in others partially successful, and are also related to age and health conditions. We suggest that the patients in our TBI series did not have a clinically distinct neurological phenotype but may have been predisposed to injury due to suboptimal nervous system function in terms of attention, risk-averse behavior, quality of threat perception and response, and neuromuscular coordination, which are known to be impaired in nervous system disorders [18,66,67]. This view is consistent with the results of this study on the presence of QVs in neurological genes in independent non-neuro and non-TBI samples. On the other hand, the prevalence of rare HI variants in intolerant neurological genes in TBI compared to non-TBI patients may generally reflect the higher damaging potential of HI variants compared to rare missense variants, even if the latter are predicted to be pathogenic and occurred in intolerant to missense variant genes. This finding indirectly supports a possible genetic predisposition to TBI.

The results of the study may contribute to the development of genetic strategies to identify genes and gene networks involved in CNS recovery. The approach of considering the cumulative risk of functional genetic variants, especially HI variants in genes intolerant to these variants, seems biologically justified, as disease severity correlates with the number of rare variants, suggesting a global role of genetic background in phenotypic heterogeneity [33,68]. As molecular genetic testing becomes more commonplace in everyday healthcare, these insights could be used to develop more tailored rehabilitation strategies.

This study has limitations. Limitations include the size of the series and patient groups, as well as their heterogeneity in terms of age, gender, severity of TBI, emergency treatment, and subsequent medical care. (1) Sample size limitations may have resulted in type I error, i.e., false positives and an inability to reproduce the results in other patient samples. (2) The sample size and heterogeneity may also be a source of type II error, leading to failure to detect results that are highly likely to be true, such as gender differences and differences in rehabilitation outcomes. (3) The small sample size, study demographic specificity, and exclusion of certain patient groups limit the applicability of the results. Furthermore, due to the highly complex and multifactorial phenotype of msTBI, we cannot rule out selection and response bias. Given these limitations, we consider our study to be a pilot study and the results to be preliminary and based on hypotheses that need to be tested in other studies and in larger samples.

## 5. Conclusions

Our exome-based study of the series of 50 patients with msTBI is the first in the context of the problem at hand and the design used. The concordant results from the three independent groups of patients allows us to suggest the existence of a possible genetic predisposition to msTBI, associated in particular with rare functional variations in intolerant genes, with a prevalent dominant mode of inheritance and neurological manifestations in the genetic phenotypes. Based on the results of this study, we plan to perform the following future studies in a larger sample of patients: (1) to search for genetic factors that may affect rehabilitation outcomes in TBI, (2) to search for genetics underlying gender differences in TBI occurrence and recovery, (3) to search for predisposing and outcome-related genetic factors in TBI patients with psychiatric and developmental disorders to delineate genetic factors contributing to TBI and these disorders, and (4) to study extreme phenotypes, as these have a more pronounced genetic predisposition, i.e., to study patients who have TBI after full recovery from a previous TBI.

This exploratory sequencing study of genetic susceptibility to TBI has yielded promising preliminary results that can be considered a first step, but not a definitive answer, to the question of genetic influences on the occurrence of TBI. Further research is warranted.

## Figures and Tables

**Table 1:** Characteristics of patients hospitalized for TBI consequences.

Characteristics	Total Group (*n* = 50)	Group 1 Discharged/ Transferred with Improvement (*n* = 25)	Group 2 Discharged/ Transferred Unchanged (*n* = 18)	Group 3 Transferred with Deterioration (*n* = 2) or Deceased (*n* = 5)
Gender and age				
Male	39 (0.78)	19 (0.76)	13 (0.72)	7 (1.00)
Female	11 (0.22)	6 (0.24)	5 (0.28)	0 (0.00)
Age	46.88 ± 18.40	47.08 ± 14.49	43.56 ± 20.90	54.71 ± 21.33
Min–max	18–86	18–79	19–86	26–82
External cause				
Falls	23 (0.46)	11 (0.44)	9 (0.50)	3 (0.42)
Transport accidents	14 (0.28)	6 (0.24)	6 (0.33)	2 (0.29)
Assault	3 (0.06)	2 (0.08)	1 (0.06)	0 (0.00)
Events of undetermined intent	10 (0.20)	6 (0.24)	2 (0.11)	2 (0.29)
Types of TBI				
Closed brain injury	39 (0.78)	19 (0.76)	14 (0.78)	6 (0.86)
Penetrating brain injury	11 (0.22)	6 (0.24)	4 (0.22)	1 (0.14)
Diffuse axonal injury	8 (0.16)	3 (0.12)	5 (0.28)	0 (0.00)
Skull fracture	26 (0.52)	11 (0.44)	12 (0.67)	3 (0.42)
Contusion	43 (0.86)	21 (0.84)	16 (0.89)	6 (0.86)
Hematoma	34 (0.68)	17 (0.68)	12 (0.67)	5 (0.71)
Hemorrhage	27 (0.54)	13 (0.52)	10 (0.56)	4 (0.57)
Surgical interventions after TBI				
Surgical interventions	35 (0.7)	16 (0.64)	14 (0.78)	5 (0.71)
Time to admission to rehab, time in rehab				
Days before admission to rehab	46.88 ± 18.40	46.42 ± 48.33	37.89 ± 32.43	113.14 ± 171.22
Min–max	10–526	9–205	10–138	11–526
Days in the rehab	41.44 ± 36.49	34.64 ± 11.72	53.39 ± 54.72	35.00 ± 27.28
Min–max	8–216	22–69	22–216	8–88
Complications/comorbidities				
Neoplasms	3 (0.06)	2 (0.08)	1 (0.06)	0 (0.00)
Hemic and lympatic/immune ^a^	35 (0.7)	15 (0.6)	15 (0.83)	5 (0.71)
Endocrine, nutritional and metabolic ^b^	27 (0.54)	10 (0.4)	11 (0.61)	6 (0.86)
Mental ^c^	37 (0.74)	19 (0.76)	12 (0.67)	6 (0.86)
Nervous ^d^	36 (0.72)	18 (0.72)	12 (0.67)	6 (0.86)
Eye ^e^	50 (1)	25 (1)	18 (1)	7 (1.00)
Ear	7 (0.14)	7 (0.28)	0 (0)	0 (0.00)
Circulatory ^f^	31 (0.62)	16 (0.64)	9 (0.5)	6 (0.86)
Respiratory ^g^	43 (0.86)	19 (0.76)	17 (0.94)	7 (1.00)
Digestive ^h^	23 (0.46)	8 (0.32)	9 (0.5)	6 (0.86)
Skin and subcutaneous tissue ^i^	26 (0.52)	11 (0.44)	10 (0.56)	5 (0.71)
Musculoskeletal/connective tissue	6 (0.12)	2 (0.08)	3 (0.17)	1 (0.14)
Genitourinary ^j^	40 (0.8)	20 (0.8)	14 (0.78)	6 (0.86)
Clinical and laboratory findings				
Dysphagia	30 (0.6)	11 (0.44)	14 (0.78)	5 (0.71)
Dysarthria and anarthria	6 (0.12)	5 (0.2)	1 (0.06)	0 (0.00)
Dysphasia and aphasia	4 (0.08)	3 (0.12)	1 (0.06)	0 (0.00)
Systemic inflammatory response syndrome of infectious origin with organ failure (severe sepsis)	9 (0.18)	1 (0.04)	3 (0.17)	5 (0.71)

**Notes.** Categorical data are reported as *n* and proportions (in parentheses); quantitative data are reported as mean and SD. The most common diseases within the classification: ^a^ iron deficiency anemia, *n* = 27; ^b^ protein-energy malnutrition, *n* = 9; ^c^ disorders due to brain damage and dysfunction, *n* = 37; ^d^ paralytic syndromes, *n* = 24; ^e^ retinopathy and retinal vascular changes, *n* = 48; ^f^ deep vein thrombosis, *n* = 23; ^g^ nosocomial pneumonia, *n* = 34; ^h^ antibiotic-associated diarrhea, *n* = 7; ^i^ decubitus ulcer, *n* = 21; ^j^ flaccid neuropathic bladder, *n* = 34.

## Data Availability

Data presented in this study are only available upon request from the corresponding author due to privacy or ethical restrictions.

## Supplementary Materials

The following supporting information can be downloaded at: https://www.mdpi.com/article/10.3390/cimb46090616/s1, Table S1: Description of scales used in the study. Table S2: Pre- and post-rehabilitation scores according to the brain injury assessment tools and the SOFA scale. Results for the total sample and for the study groups are presented as mean and SD, with min and max values in parentheses. Superscript indices indicate the number of patients with data for calculations. The last column contains statistical data. The assessment of the normality of the distribution showed heterogeneous results; therefore, both Pearson and Spearman correlation coefficients are provided (the data are shown on the gray shaded lines), and the number of patients is provided in brackets. The Kruskal–Wallis test was used to compare all three groups, and the MWU test was used to compare whether there were differences between two independent groups (group 1 and group 2). Nominally significant *p*-values are shown in bold. Significant *p*-values after FDR correction (separately for correlation tests and statistical hypothesis testing) are marked with an asterisk. Table S3: QVs in intolerant genes annotated for each TBI patient. Table S4: Enrichr results in Reactome 2022 and GO Biological Process 2023 modules for all genes with rare HI and missense variants (REVEL > 0.5) and for intolerant genes with these variants in a series of 50 patients. Table S5: Enrichr results in Reactome 2022 and GO Biological Process 2023 modules for intolerant genes with QVs in three patient groups. Table S6: Annotation of intolerant genes with QVs. Table S7: QVs in intolerant genes annotated for each of 50 age- and sex-matched patients (with COVID-19) without TBI. The sample of non-TBI patients included 32 males and 18 females aged 51.14 ± 16.28 years (mean ± SD). Figure S1: Number of intolerant genes with QVs per patient in the male and female patient groups.
