# Supplementary figures and images for: Genetics and Traumatic Brain Injury: Findings from an Exome-Based Study of a 50-Patient Case Series
Source: Curr Issues Mol Biol. 2024 Sep 17;46(9):10351–68. doi: 10.3390/cimb46090616 (PMC11430351; doi:10.3390/cimb46090616)

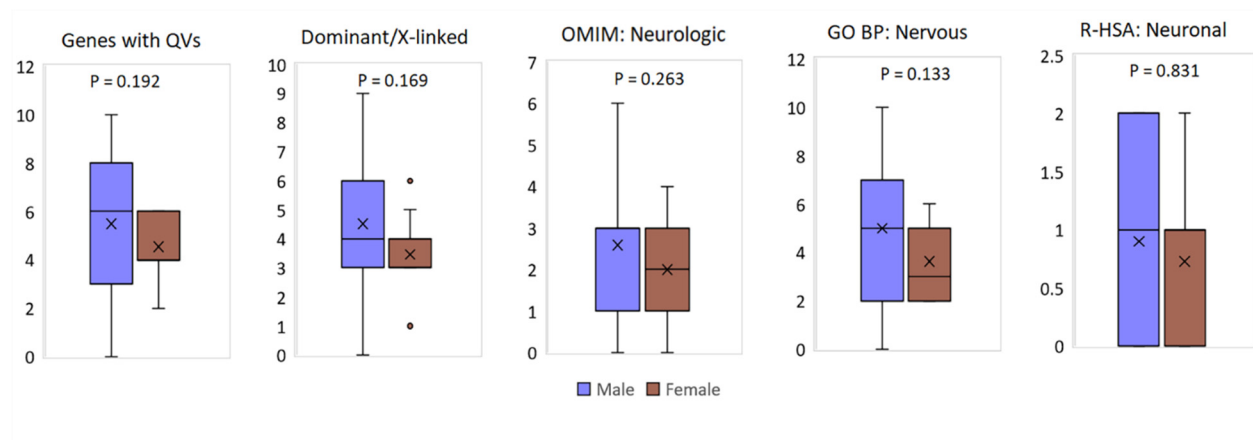

Figure S1. Number of genes with QVs per patient in the male and female patient groups.

Supplement: Supplementary file 1 [file cimb-46-00616-s001.zip › Figure S1.pdf]
